# Abdominal Tuberculosis Presenting as an Umbilical Sinus: A Case Report

**DOI:** 10.7759/cureus.31184

**Published:** 2022-11-07

**Authors:** Rishwanth Vetri, Vishmita Kannichamy, Vinni Anna Jacob, Surabhi Sainath

**Affiliations:** 1 General Surgery, Stanley Medical College, Chennai, IND

**Keywords:** umbilical pus discharge, abdominal distention, peritoneal tuberculosis, umbilical sinus, abdominal tuberculosis

## Abstract

Abdominal tuberculosis, a form of extrapulmonary tuberculosis is common in developing countries like India. Manifestations of abdominal tuberculosis are widely variable. Its incidence is high among human immunodeficiency virus (HIV) infected patients in the adult population.

Here, we report a 26-year-old male, initially treated in a private clinic for abdominal distention, loss of appetite, and loss of weight for one month. Magnetic resonance imaging (MRI) of the abdomen showed a large loculated fluid collection. Computed tomography (CT) of the chest showed signs of pulmonary tuberculosis. The patient was started on anti-tuberculosis therapy (ATT) and discharged. Ten days later, the patient presented to our hospital with foul-smelling pus discharging from the umbilicus and necrosis of the surrounding tissue. Abdominal examination revealed a tense, distended, and tender abdomen. The patient was referred for emergency laparotomy. The intraoperative findings showed features that were a combination of plastic fibrous type and encysted loculated type of peritoneal tuberculosis.

The above-mentioned case describes a very uncommon presentation of abdominal tuberculosis. A high degree of suspicion is required for diagnosing such conditions, especially in immunocompromised individuals. The case report also highlights the difficulties in the diagnosis of abdominal tuberculosis.

## Introduction

Tuberculosis remains a major burden globally with an estimated death of 1.5 million among the 10 million affected in 2020. Tuberculosis affects all age groups and is present in all countries, predominantly developing countries like India. India in 2020 accounted for the maximum number of cases followed by China [1]. The estimated incidence of tuberculosis in India was found to be 312 per 100,000 population in 2021 [2]. Indoor air pollution, low body mass index (BMI), and smoking were found to be the major risk factors for TB infection in India [1]. Abdominal tuberculosis is the sixth most common presentation of extrapulmonary tuberculosis, most commonly seen in human immunodeficiency virus (HIV) infected patients in the adult population [3,4]. The association of abdominal tuberculosis with an umbilical sinus and associated tissue necrosis is very rare. This case report highlights the case of a 26-year-old male presenting with complaints of foul-smelling pus discharging from the umbilicus where the findings were consistent with abdominal peritoneal tuberculosis.

## Case presentation

We report a 26-year-old male, initially treated in a private clinic for abdominal distention, loss of appetite, and loss of weight for one month. There was no history of prolonged fever, night sweats, or chronic cough. There was no significant contact history and no history of similar complaints among the family members. The patient was of a low socioeconomic class and lived in a crowded household. The patient had no other significant history or comorbid history.

**Figure 1:**
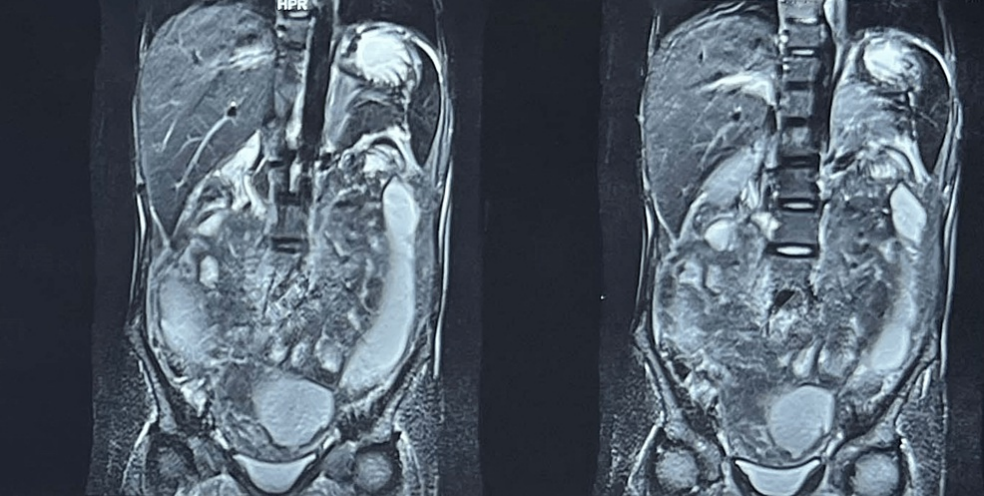
MRI of the abdomen showing signs of hollow viscus perforation

Magnetic resonance imaging (MRI) of the abdomen was taken in the private clinic which showed a large loculated fluid collection wherein the diagnosis was strongly suggestive of hollow viscus perforation. Figure 1 shows an MRI of the abdomen with signs suggestive of hollow viscus perforation. The patient deferred any further treatment and was discharged upon request.

**Figure 2:**
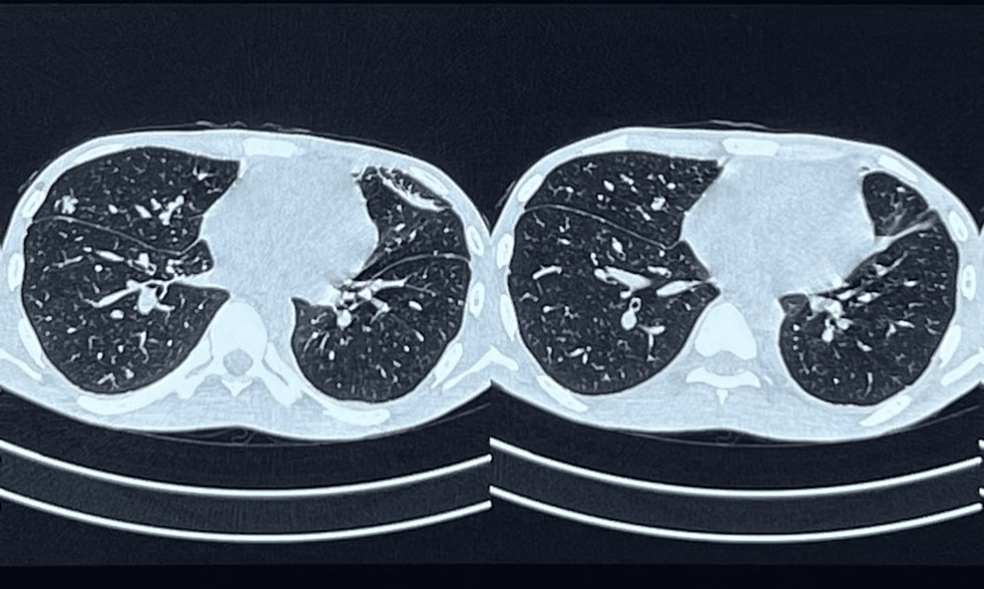
CT of the chest showing multiple ill-defined nodules

Two weeks later, the patient presented to the same private clinic with similar complaints. The following tests were done due to raised suspicion of abdominal tuberculosis. An ultrasonogram-guided ascitic fluid aspirate was taken for protein and Adenosine Deaminase (ADA) level analysis, which revealed an elevated ADA level of 282 U/l. The patient was subjected to computed tomography (CT) of the chest which showed multiple randomly distributed ill-defined nodules, left-sided minimal pleural effusion, and right-sided loculated pneumothorax (Figure 2). Findings were consistent with pulmonary tuberculosis. CT of the abdomen showed the large loculated fluid collection and air pockets in the peritoneal cavity which was compressing and displacing the bowel loops peripherally suggestive of hollow viscus perforation.

The patient was started on anti-tuberculosis therapy (ATT) but was not compliant with medication because of several episodes of loose stools. Ten days later, he presented to our hospital with complaints of abdominal pain and constipation for the past one day. There was foul-smelling pus discharging from the umbilicus and necrosis of the tissue surrounding the umbilicus.

**Figure 3:**
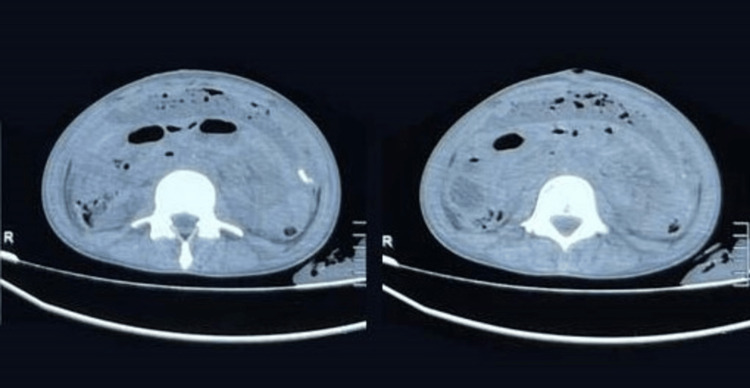
CT of the abdomen showing moderate ascites with multiple air pockets in the peritoneal cavity

On examination, a febrile, moderately built, and poorly nourished male, dehydrated with the following vitals, pulse rate 110/min, blood pressure 110/70 mmHg, respiratory rate 22/min, a saturation of peripheral oxygen 98% in room air. The patient weighed 29 kg with an abdominal girth of 63 cm. There was no generalized lymphadenopathy. Abdominal examination revealed a tense, distended, and tender abdomen. Along with pus discharge from the umbilical sinus, necrosis was seen in the tissue surrounding the umbilicus. No bowel sounds were heard and shifting dullness was present. CT of the abdomen was taken, which showed ascites with multiple foci of air pockets in the peritoneal cavity (Figure 3). 

**Figure 4:**
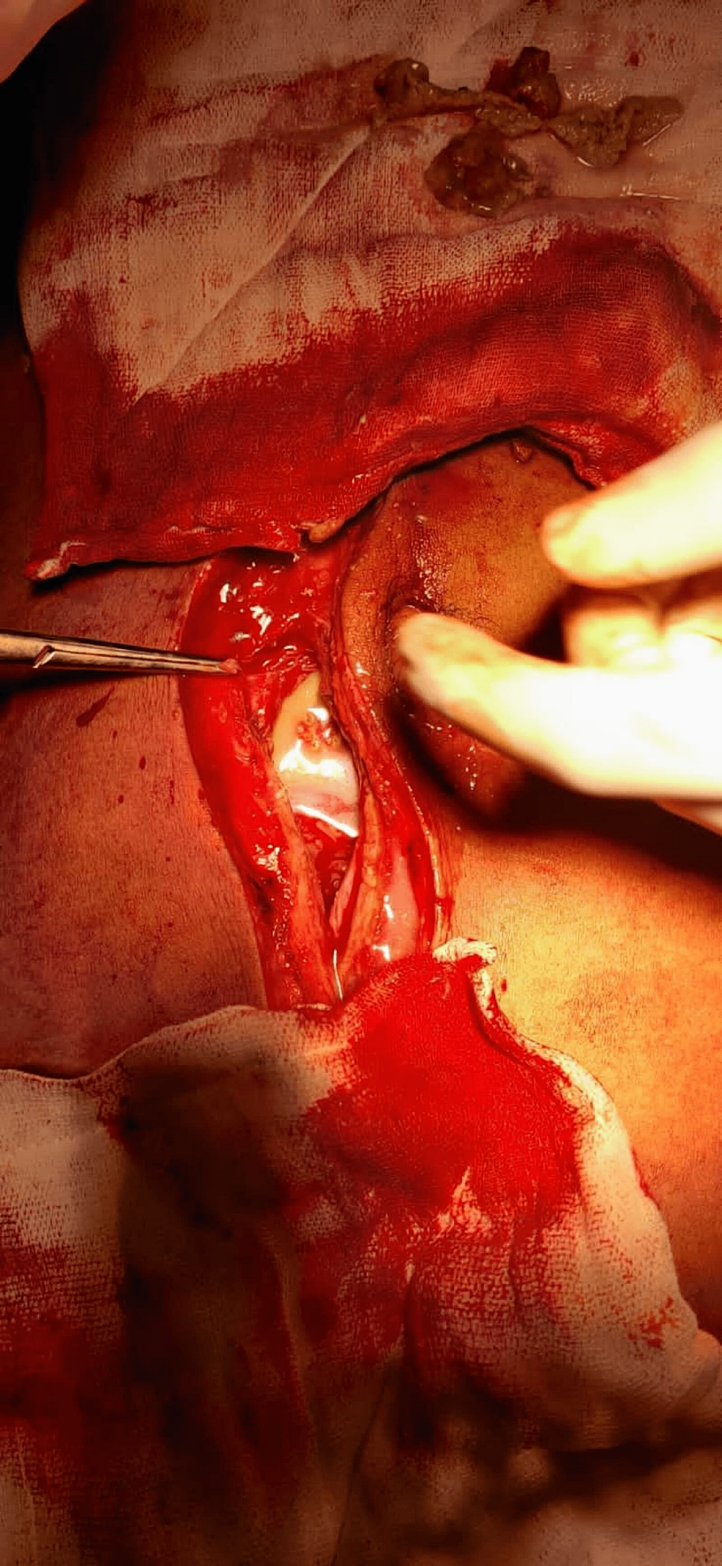
Loculated pus in the peritoneal cavity

**Figure 5:**
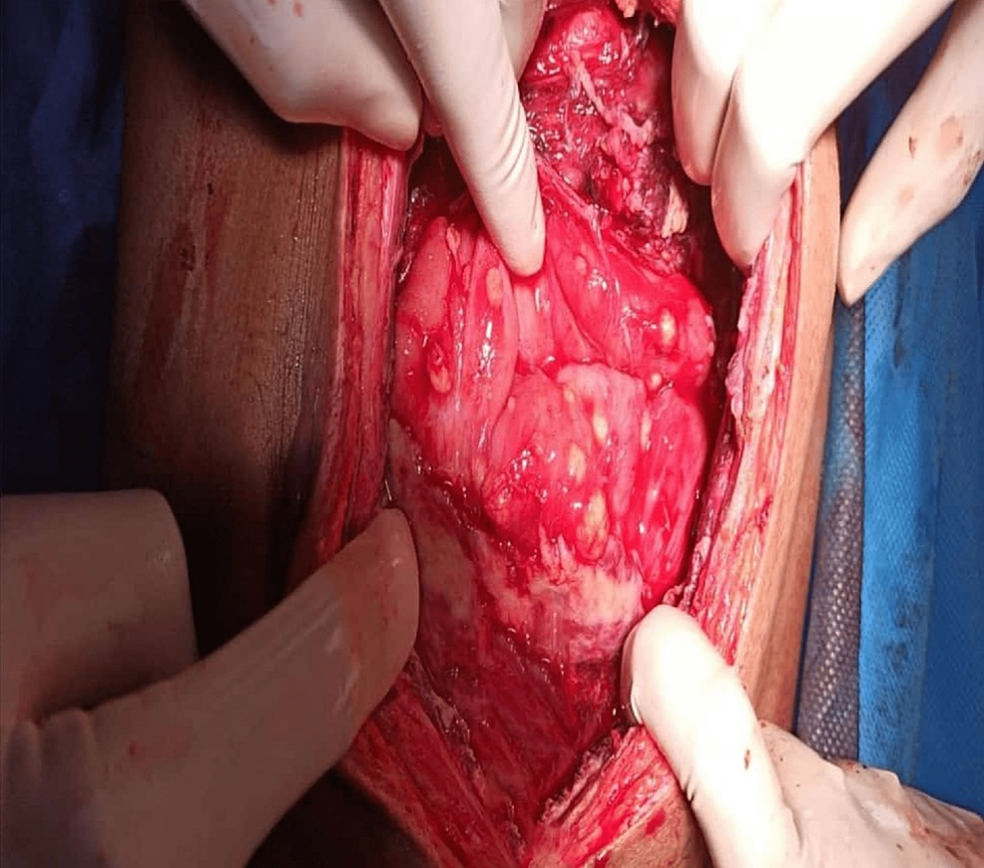
Multiple serous tubercles with dense adhesions

**Figure 6:**
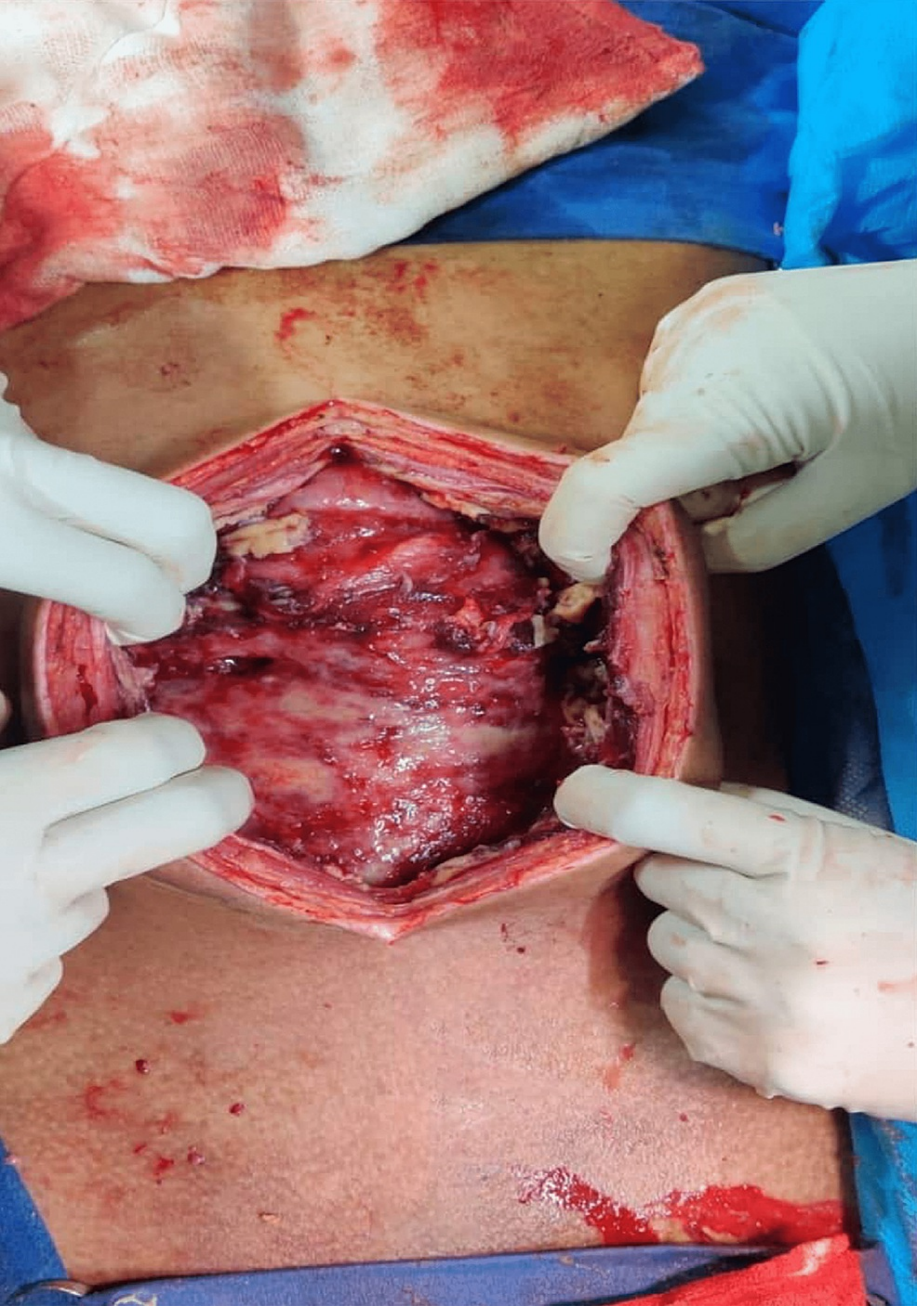
Findings show partially digested food particle and dense adhesions

The patient was referred for emergency laparotomy. Figure 4 shows pyo-peritoneum, and pus of about 350-400 mL was drained from the peritoneal cavity with flakes. Figure 5 shows thickened peritoneum with the formation of an “abdominal cocoon,” dense adhesions between bowel loops, and multiple serous tubercles noted. The findings showed features of a combination of plastic fibrous type and encysted loculated type. Furthermore, a large loculated purulent collection of pus with partially digested food particles was present, indicating a concealed perforation. The site of perforation could not be delineated due to dense fibrous adhesions. Figure 6 shows partially digested food particles and fibrous adhesions. 

 

 

After a thorough wash with normal saline, thickened peritoneum and serous nodules were biopsied and sent for histopathology. Two drainage tubes (DTs) were placed because the site of perforation could not be identified, one in the pelvis and the other in the hepatorenal pouch. Histopathological findings showed granulomatous inflammation with areas of necrosis which was confirmatory of the diagnosis made earlier. The patient was started on empirical antibiotics and later changed to third-generation cephalosporins according to the pus culture sensitivity report obtained from the pus in the pelvic DT. A cartridge-based nucleic acid amplification test (CBNAAT) of ascitic fluid was done, and Mycobacterium tuberculosis (MTB) was detected and was found to be sensitive to rifampicin. The patient was started on ATT for eight months. Table 1 shows the drug regimen followed by the patient. It consists of a two-month intensive phase with five drugs (isoniazid, rifampicin, pyrazinamide, ethambutol, and streptomycin) followed by one month of four drugs (isoniazid, rifampicin, pyrazinamide, and ethambutol). This is followed by five months of continuation phase with three drugs (isoniazid, rifampicin, and pyrazinamide). 

**Table 1:** Drug regimen followed by the patient for abdominal tuberculosis

	Drug	Dosage
Intensive Phase		
1^st^ and 2^nd^ month (2 months)	Isoniazid (H) Rifampicin (R) Pyrazinamide (Z) Ethambutol (E) Streptomycin (S)	300 mg(H), 450 mg(R), 1500mg(Z), 750 mg(E), 450 mg(S)
3^rd^ month (1 month)	Isoniazid (H) Rifampicin (R) Pyrazinamide (Z) Ethambutol (E)	300 mg(H), 450 mg(R), 1500mg(Z), 750 mg(E)
Continuation Phase		
4^th^ -8^th^ month (5 months)	Isoniazid (H) Rifampicin (R) Pyrazinamide (Z)	300 mg(H), 450 mg(R), 1500mg(Z)

The patient was started on ATT after two days of recovery. The post-operative period was uneventful, and the patient recovered well from surgery. A one-year follow-up of the patient was done, and the scar was found to be healed adequately. The patient had no other complaints.

## Discussion

Abdominal tuberculosis may involve any site along the Gastrointestinal (GI) tract, peritoneum, mesentery, or abdominal lymph nodes. Although rare, it may also affect organs such as the liver, spleen, and pancreas [4]. The most common age group affected is 25-45 years. The mode of spread of infection is either by ingestion (food contaminated with bacilli/sputum containing bacilli), hematogenous spread from the lungs, lymphatic spread, or retrograde spread from the fallopian tubes to involve the peritoneum [5]. Due to the abundance of Peyer’s patches and stasis of luminal contents, the ileocecal junction is the most commonly reported site for abdominal tuberculosis.

Peritoneal tuberculosis is not uncommon, factors such as migration, the epidemic of AIDS, and an increase in the use of immunosuppressive therapy have led to its resurgence in recent years [6]. The diagnosis of this condition is difficult owing to its variability of presentation, non-specific radiologic signs, vague symptoms, and the insidious nature of the disease [5,7].

The presentation of abdominal tuberculosis may be acute or chronic. Patients with intestinal tuberculosis commonly present with colicky abdominal pain, abdominal distention, vomiting, constipation, and diarrhea. In peritoneal tuberculosis the patient presents with abdominal distention, subacute intestinal obstruction, diffuse abdominal pain, recurrent fever, or loss of appetite and weight, some patients remain asymptomatic [8]. Our patient had typical features suggestive of peritoneal tuberculosis. Diagnosis is often delayed due to the non-specificity of the symptoms and similarity of the symptoms to other GI conditions, this leads to increased mortality [4]. Rare instances have been documented where tubercular granulomatous hepatitis presented as painless fluctuating jaundice and anemia [9]. Hence, a high degree of suspicion is required for an appropriate diagnosis. Tuberculous peritonitis should be considered as a differential in all patients with serum-ascites albumin gradient less than 11 g/L [10]. Various investigations such as an ultrasonogram (USG) of the abdomen, an abdominal radiograph (shows calcifications and perforation if present), a chest radiograph (to find out the primary focus), CT abdomen, barium study x-ray (barium meal/barium enema), colonoscopy followed by polymerase chain reaction (PCR) of the biopsied tissue, laparoscopy, ascitic tap fluid analysis (protein and ADA level analysis), transabdominal peritoneoscopy (to visualize peritoneum and collection of ascitic fluid) etc. aids in the diagnosis. Yet, a single confirmatory test to make a definitive diagnosis is not available. CT is the most prevalent imaging modality used in the majority of studies [11,12]. Currently, laparoscopy with a biopsy of the specimen is the preferred investigation [12,13]. However, the measurement of ADA levels in the ascitic fluid provides an equally valuable insight [14] and has aided in the diagnosis of our case. Despite this, in many instances, the diagnosis is made intra-operatively.

In peritoneal tuberculosis, enormous thickening of the parietal peritoneum occurs, and dense adhesions between the peritoneum and with small intestine as the content inside look like an “abdominal cocoon.” Peritoneal tuberculosis is classified into three main types: wet ascitic, fixed fibrotic, and dry plastic [15]. However, this classification is often not adequate, as patients usually present with a combination of features. In our case, the patient presented with features suggestive of the fixed fibrotic and dry plastic types. In the former type, the ascites get loculated because of fibrinous deposition, and dullness which is not shifting is the characteristic feature, although not present in our patient. Widespread adhesions between coils of the intestine thickened parietal peritoneum and bowel distention are characteristic of the dry plastic type of parietal tuberculosis. Six months of ATT is sufficient except in cases of drug-resistant cases and newly diagnosed cases of extrapulmonary TB, surgical intervention may be required for complications such as bowel perforation, and fistula formation [16]. The role of corticosteroids in treatment remains controversial [8]. This case report talks about a rare case of abdominal tuberculosis presenting with an umbilical sinus with surrounding tissue necrosis.

## Conclusions

We report a rare case of abdominal tuberculosis presenting as a case of discharging umbilical sinus with surrounding tissue necrosis. It further elaborates on the difficulties of diagnosing a case of abdominal tuberculosis. Due to the lack of a definitive diagnostic method, a combination of techniques is required to arrive at a diagnosis. An insufficient uniform classification system for peritoneal tuberculosis also hinders the management of patients. A high degree of suspicion should be maintained for appropriate diagnosis, especially in immunocompromised individuals.
